# A Captive

**Published:** 1892-12

**Authors:** 


### A CAPTIVE.

There's magic in thy glance,
Fair maid;
A witchery to entrance;
He said.
"I own the fateful charm,
yield me, I disarm,"
He said.

"No knight I captive take
Self-made;
You must your ransom make,"
She said.
"Besides, what use my charms,
If you've laid down your arms,"
She said. —G. B. L.

